# Risk of schizophrenia, schizoaffective, and bipolar disorders by migrant status, region of origin, and age-at-migration: a national cohort study of 1.8 million people

**DOI:** 10.1017/S0033291718003227

**Published:** 2018-12-05

**Authors:** Jennifer Dykxhoorn, Anna-Clara Hollander, Glyn Lewis, Cecelia Magnusson, Christina Dalman, James B Kirkbride

**Affiliations:** 1 Division of Psychiatry, UCL, London, UK; 2 Department of Public Health Sciences, Karolinska Institutet, Stockholm, Sweden; 3 Centre for Epidemiology and Community Medicine, Stockholm County Council, Stockholm, Sweden

**Keywords:** Bipolar disorder, epidemiology, mania, migration, psychotic disorders, schizophrenia

## Abstract

**Background:**

We assessed whether the risk of various psychotic disorders and non-psychotic bipolar disorder (including mania) varied by migrant status, a region of origin, or age-at-migration, hypothesizing that risk would only be elevated for psychotic disorders.

**Methods:**

We established a prospective cohort of 1 796 257 Swedish residents born between 1982 and 1996, followed from their 15th birthday, or immigration to Sweden after age 15, until diagnosis, emigration, death, or end of 2011. Cox proportional hazards models were used to model hazard ratios by migration-related factors, adjusted for covariates.

**Results:**

All psychotic disorders were elevated among migrants and their children compared with Swedish-born individuals, including schizophrenia and schizoaffective disorder (adjusted hazard ratio [aHR]_migrants_: 2.20, 95% CI 1.96–2.47; aHR_children_ : 2.00, 95% CI 1.79–2.25), affective psychotic disorders (aHR_migrant_1.42, 95% CI 1.25–1.63; aHR_children_: 1.22 95% CI 1.07–1.40), and other non-affective psychotic disorders (aHR_migrant_: 1.97, 95% CI 1.81–2.14; aHR_children_: 1.68, 95% CI 1.54–1.83). For all psychotic disorders, risks were generally highest in migrants from Africa (i.e. aHR_schizophrenia_: 5.24, 95% CI 4.26–6.45) and elevated at most ages-of-migration. By contrast, risk of non-psychotic bipolar disorders was lower for migrants (aHR: 0.58, 95% CI 0.52–0.64) overall, and across all ages-of-migration except infancy (aHR: 1.20; 95% CI 1.01–1.42), while risk for their children was similar to the Swedish-born population (aHR: 1.00, 95% CI 0.93–1.08).

**Conclusions:**

Increased risk of psychiatric disorders associated with migration and minority status may be specific to psychotic disorders, with exact risk dependent on the region of origin.

## Background

The relationship between migration and schizophrenia risk is well-established (Ødegaard, 1932; Cantor-Graae and Selten, 2005; Fearon and Morgan, 2006; Bourque *et al*., 2011; Cantor-Graae and Pedersen, 2013; Close *et al*., 2016), and extends to children of migrants (Cantor-Graae and Selten, 2005; Bourque *et al*., 2011). The risk appears higher among visible minorities, such as the black Caribbean and African populations in the UK (Fearon *et al*., 2006; Kirkbride *et al*., 2012*b*), and is not explained by differences in age, sex, or socioeconomic position (Kirkbride *et al*., 2008). By contrast, elevated rates have not been consistently shown for bipolar disorder among migrants (Swinnen and Selten, 2007) or their children (Cantor-Graae and Pedersen, 2013; Pignon *et al*., 2017). Despite this, previous studies have rarely distinguished between bipolar disorders presenting with and without psychotic features. When restricted to those with psychosis, there is some evidence of higher rates of bipolar disorder in migrants and their children (Lloyd *et al*., 2005).

The impact of migration and social adversity on mental disorders also varies over the life course (Patel and Goodman, 2007), although only limited, equivocal research has investigated the role that age-at-migration may play in shaping risk (Veling *et al*., 2011; Pedersen and Cantor-Graae, 2012; Kirkbride *et al*., 2017*b*). For example, a Dutch study found the highest risk of psychotic disorders with infant migration (Veling *et al*., 2011), while a more recent British study found risk peaked with early childhood migration (Kirkbride *et al*., 2017*b*). Finally, a Danish study found no association between age-at-migration and schizophrenia (Pedersen and Cantor-Graae, 2012). No study to date has examined age-at-migration in relation to bipolar disorders, with or without psychosis.

To clarify these issues, we used national register data from Sweden, a country of 10 million people with a long history of immigration (OECD, 2017), to investigate whether migrant status, region of origin, and age-at-migration acted specifically on the risk of developing psychotic disorders, including schizophrenia, affective psychoses (i.e. bipolar disorder with psychotic symptoms or psychotic depression), or also extended to non-psychotic bipolar disorder and mania. Given the earlier research, we hypothesized that excess risk in migrant groups would be limited to psychotic disorders. Similarly, we hypothesized that migration during infancy and early childhood would increase psychosis risk, but not non-psychotic bipolar disorder and mania. These periods of the life course are marked by rapid social, neurobiological, and cognitive development, including maturation of the dopaminergic system in the prefrontal cortex during infancy (Rothmond *et al*., 2012; Selemon and Zecevic, 2015) and development of ‘theory of mind’ and general cognition during early childhood (Perner and Lang, 1999; Garety *et al*., 2001; Colvert *et al*., 2008). Migration during such periods may disrupt typical neurocognitive development, which subsequently increases psychosis risk. Whilst cognitive deficits have been observed in both schizophrenia and bipolar disorder, cognitive impairments in bipolar disorder appear less severe (Trotta *et al*., 2014), and other developmental abnormalities also show specificity towards psychotic disorders during this period (Reichenberg *et al*., 2002; Hill *et al*., 2013).

## Methods

### Study design and population

We used multiple national Swedish registers to identify all individuals born between 1982 and 1996, living in Sweden after their 15th birthday, including those who immigrated to Sweden. Cohort entry was age 15 years, or date of first immigration to Sweden, if later. Individuals were followed from cohort entry (earliest: 1 January 1997) until 31 December 2011, or exit from the cohort due to the diagnosis of a psychiatric disorder of interest (see below), emigration or death, whichever came sooner. The Cause of Death register was used to obtain the date of death and the Migration Register was used to record date of migration. Individuals who died emigrated for the final time, or who were diagnosed with a psychiatric outcome before age 15 were excluded.

### Outcomes

We linked participants to the National Patient Register to determine a psychiatric diagnosis in either inpatient or outpatient settings according to the International Classification of Diseases, 10th revision [ICD-10]. We studied four psychiatric outcomes: schizophrenia or schizoaffective disorder (F20.X, F25.X); affective psychotic disorders (F30.2, F31.2, F31.5, F32.3, F33.3), other non-affective psychotic disorders (F21.X-F24.X, F28.X- F29.X) and; bipolar disorder or manic symptoms without confirmed psychotic symptoms (F30.1, F30.8, F30.9, F31.0–31.1, F31.3–31.4, F31.6–31.9). For individuals who received diagnoses on multiple visits to in- or outpatient services, we adopted a hierarchal classification system, informed by clinical expertise (GL, CD, CM, AH) and consistent with earlier research (Björkenstam *et al*., 2013), as follows: schizophrenia or schizoaffective disorder, affective psychotic disorders, other non-affective psychotic disorders, and finally, non-psychotic bipolar disorder or mania; date of cohort exit was defined as the date at which they received this diagnosis.

### Exposures

We defined migrant status according to information in the Total Population and Multi-Generational registers. Participants were classified as (i) *migrants* if they were born outside of Sweden; (ii) *children of migrants* if they were born in Sweden with one or both parents born outside of Sweden, and; (iii) *Swedish-born* if they were born in Sweden to two Swedish-born parents. The region of origin was defined by Statistics Sweden as Sweden, Finland, other Nordic countries, other European countries, Asia and Oceania, the Middle East, Africa, North America, South America, and unknown, based on country of birth. For migrants, we categorized age-at-first-migration into five groups: infancy (0–2 years), early childhood (3–6 years), middle childhood (7–12 years), adolescence (13–18 years) and early adulthood (19–29 years). We considered sex, current age, and follow-up period (see below) as *a priori* confounders. We also controlled for income in supplemental analyses, obtained from the Longitudinal Integration Database for Health Insurance and Labour Market Studies [LISA]. Since 1990, LISA has estimated total disposable family income from all sources each year (salary, wages, welfare, pensions), weighted for family size. As age 16 is the earliest an individual is included in the LISA, for most participants, we utilized information on their family income at age 15 via linkage to their parents, including adoptive parents where relevant. For migrants arriving in Sweden after age 15, we included their family income as first recorded in the LISA database. Where no income could be estimated, participants were excluded from these analyses (see below). We calculated income quintiles in each year for the entire population and assigned this value to participants in the year of their cohort entry. This method implicitly takes income inflation into account.

### Statistical analysis

We first generated descriptive characteristics of the sample. Next, for each outcome, we investigated whether incidence varied by migrant status, region of origin, and age-at-migration using Cox proportional hazard regression. We estimated unadjusted and adjusted hazard ratios [aHR] and 95% confidence intervals [95% CI] for each exposure. To account for possible period effects (e.g., due to possible changes in health care services over the 14-year follow-up period), we split the data into 5-year bands of calendar time (1997–2001, 2002–2006, 2007–2011), modelled as a time-varying covariate. We also modelled age as a time-varying covariate (15–17, 18–20, 21–23, 24–26, 27–30 years), given risk of psychiatric disorders varies substantially by age (Hollander *et al*., 2016; Kirkbride *et al*., 2017*a*). The proportional hazards assumption was evaluated using Schoenfeld residuals and examination of log-log plots. We conducted two sensitivity analyses. First, because income may have been on the causal pathway between migration-related exposures and risk of psychiatric disorders, these adjustments were presented as sensitivity analyses, excluding those with missing information on this variable. Second, we included a washout period to exclude migrant participants diagnosed with an outcome of interest within 2 years of immigration to Sweden, who may have been prevalent cases. All modelling was conducted in Stata, version 13.

## Results

We identified 1 796 257 individuals who contributed over 12.79 million person-years of follow-up (Table 1). Of these, 17.8% were migrants and 11.2% were children of migrants. In all, 15 423 individuals (0.9%) were diagnosed with an outcome of interest during follow-up, including 2172 individuals (0.1%) with schizophrenia or schizoaffective disorder, 2163 (0.1%) with an affective psychotic disorder, 4510 (0.3%) with another non-affective psychotic disorder, and 6295 (0.4%) with non-psychotic bipolar disorder. Among cases, the proportion of women diagnosed with a psychiatric disorder varied by migrant status (χ^2^ on 2 degrees of freedom [*df*]; *p* < 0.001), being highest amongst the Swedish-born population (59.2%) and lowest amongst migrants (45.0%). As expected, migrants with psychiatric diagnoses were more likely to be in the lowest income quintile (30.2%) compared to 5.3% of Swedish-born, and 8.6% of children of migrants (χ^2^ on 10 *df*; *p* < 0.001). Additional cohort characteristics are presented in Table 1.

**Table 1.** Cohort characteristics by migrant status

	Swedish-born (*n* = 1 275 748)			Children of migrants (*n* = 200 407)			Migrants (*n* = 320 102)		
	Cases	%	Total person-years	Cases	%	Total person-years	Cases	%	Total person-years
Psychiatric diagnosis									
Schizophrenia + schizoaffective	1333	0.1	9 802 814	368	0.2	1 403 948	471	0.2	1 631 366
Affective psychotic disorders	1532	0.1	9 802 240	259	0.1	1 404 455	372	0.1	1 631 982
Other non-affective psychotic disorders	2899	0.2	9 797 765	687	0.3	1 402 980	924	0.3	1 630 197
Bipolar disorder without psychotic symptoms	5130	0.4	9 793 959	724	0.4	1 403 407	441	0.1	1 632 041
Sex									
Male	4499	40.8	5 034 939	1017	48.2	720 336	1265	55.2	823 667
Female	6521	59.2	4 737 861	1094	51.8	678 348	1027	44.8	802 363
Income									
Quintile 1 (Lowest)	588	5.3	293 129	182	8.6	84 411	690	30.1	529 386
Quintile 2	2021	18.3	1 212 372	431	20.4	228 359	300	13.1	192 003
Quintile 3	2481	22.5	1 731 842	565	26.8	347 721	470	20.5	329 979
Quintile 4	2985	27.1	3 011 696	485	23.0	374 611	381	16.6	270 114
Quintile 5 (Highest)	2932	26.6	3 517 217	436	20.7	358 555	278	12.1	169 106
Unknown	13	0.1	6544	12	0.6	5026	173	7.6	135 443
Age-at-migration									
Swedish born	11 020	100.0	9 772 800	2111	100.0	1 398 684			
Infancy (0–2)							384	16.8	221 374
Early childhood (3–6)							436	19.0	297 272
Middle childhood (7–12)							539	23.5	394 417
Adolescence (13–18)							458	20.0	301 284
Early adulthood (19–29)							475	20.7	397 242
Unknown							0	0.0	14 441
Region of origin									
Sweden	11 020	100.0	9 772 800	2111	100.0	1 398 684			
Finland							59	2.6	29 770
Other Nordic							83	3.6	54 832
Europe							582	25.4	522 376
Asia + Oceania							356	15.5	278 469
Middle East							560	24.4	441 042
Africa							400	17.5	161 752
North America							66	2.9	35 259
South America							185	8.1	101 726
Unknown							1	0.0	804

**Fig. 1.**
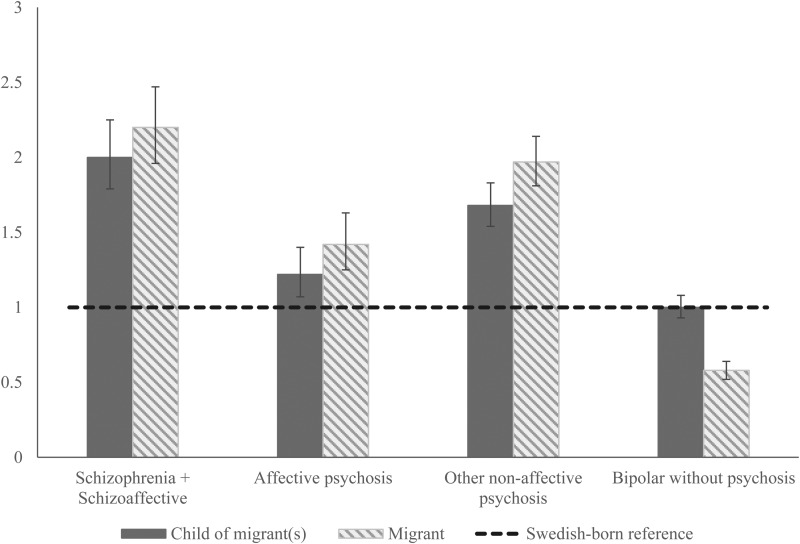
Risk of schizophrenia, schizoaffective, and bipolar disorders by migrant status, region of origin, and age-at-migration. A national cohort study of 1.8 million people. Adjusted hazard ratios by migrant status. 95% CI, 95% confidence interval. ^1^Adjusted for age, sex, and time period.

Risk of all psychotic disorders was elevated among migrants and their children compared with Swedish-born individuals, after adjustment for confounding. For example, risk of schizophrenia and schizoaffective disorder was approximately doubled in migrants (aHR: 2.20; 95% CI 1.96–2.47) and their children (aHR: 2.00; 95% CI 1.79–2.25), with similar results obtained for other non-affective psychotic disorders (Table 2). Risk of affective psychoses was also elevated amongst migrants (aHR: 1.42; 95% CI 1.25–1.63) and their children (aHR: 1.22; 95% CI 1.07–1.40), albeit to a lesser extent than for schizophrenia and other psychoses (Fig. 1). In contrast, migrants were at reduced risk of bipolar disorder without psychosis (aHR: 0.58, 95% CI 0.52–0.64) compared with the Swedish-born population, while the risk was equivocal for children of migrants (aHR: 1.00, 95% CI 0.93–1.08).

**Table 2.** Unadjusted and Adjusted hazard ratios by migrant status

	*N*	%	Unadjusted		Adjusted^a^	
			Hazard ratio	95% CI	Hazard ratio	95% CI
Schizophrenia + schizoaffective						
Swedish born (reference)	1333	61.4	1		1	
Children of migrants	368	16.9	2.00	1.78–2.25	2.00	1.79–2.25
Migrant	471	21.7	2.43	2.19–2.70	2.20	1.96–2.47
Affective psychotic disorders						
Swedish born (reference)	1532	70.8	1		1	
Children of migrants	259	12.0	1.24	1.09–1.42	1.22	1.07–1.40
Migrant	372	17.2	1.58	1.40–1.77	1.42	1.25–1.63
Other non-affective psychotic disorders						
Swedish born (reference)	2899	64.3	1		1	
Children of migrants	687	15.2	1.70	1.56–1.84	1.68	1.54–1.83
Migrant	924	20.5	2.13	1.98–2.30	1.97	1.81–2.14
Bipolar disorder without psychosis						
Swedish born (reference)	5130	81.5	1		1	
Children of migrants	724	11.5	1.02	0.95–1.11	1.00	0.93–1.08
Migrant	441	7.0	0.59	0.53–0.65	0.58	0.52–0.64

95% CI, 95% confidence interval.

a Adjusted for age, sex, and time period.

The excess risk of psychotic disorders in migrants persisted for participants from all regions of origin (Table 3). Thus, for schizophrenia and schizoaffective disorder, all migrants (except people of ‘other Nordic’ origin) were at increased risk, while other non-affective psychotic disorders were elevated for migrants from all regions of origin. For affective psychoses, migrants from Africa, South America, Asia & Oceania, and the Middle East were at increased risk. For all psychotic outcomes, risk was highest amongst migrants from Africa, including schizophrenia (aHR: 5.24, 95% CI 4.26–6.45), other non-affective psychoses (aHR: 4.39, 95% CI 3.77–5.10), and to a lesser degree, affective psychosis (aHR: 2.01, 95% CI 1.49–2.70). By contrast, migrants from Africa, Europe, the Middle East, Asia, and Oceania showed reduced risk of bipolar disorder without psychosis compared with the Swedish-born population (Table 3), and only migrants from Finland (aHR: 1.56, 95% CI 1.01–2.41) and North America (aHR: 1.69, 95% CI 1.15–2.50) had elevated risk.

**Table 3.** Unadjusted and adjusted hazard ratios by region of origin

	*N*	%	Unadjusted		Adjusted^a^	
			Hazard ratio	95% CI	Hazard ratio	95% CI
Schizophrenia + schizoaffective						
Swedish-born (reference)	1333	61.4	1		1	
Finland	7	0.3	2.26	1.07–4.75	1.90	0.90–4.02
Other Nordic	10	0.5	1.83	0.98–3.40	1.50	0.80–2.82
Europe	125	5.8	2.01	1.67–2.41	1.85	1.53–2.23
Asia + Oceania	61	2.8	1.92	1.48–2.49	1.72	1.32–2.25
Middle East	123	5.7	2.25	1.87–2.71	2.07	1.71–2.50
Africa	105	4.8	5.94	4.87–7.25	5.24	4.26–6.45
North America	11	0.5	2.77	1.53–5.02	2.44	1.34–4.43
South America	29	1.3	1.94	1.32–2.83	1.84	1.26–2.70
Affective psychotic disorders						
Swedish-born (reference)	1532	70.8	1		1	
Finland	6	0.3	1.58	0.71–3.52	1.35	0.60–3.03
Other Nordic	7	0.3	1.02	0.49–2.15	0.89	0.42–1.89
Europe	106	4.9	1.37	1.11–1.68	1.23	0.99–1.53
Asia + Oceania	71	3.3	1.76	1.37–2.25	1.53	1.18–1.99
Middle East	97	4.5	1.48	1.19–1.83	1.37	1.10–1.70
Africa	50	2.3	2.31	1.73–3.07	2.01	1.49–2.70
North America	7	0.3	1.49	0.71–3.13	1.36	0.64–2.86
South America	28	1.3	1.79	1.22–2.62	1.81	1.23–2.65
Other non-affective psychotic disorders						
Swedish-born (reference)	2899	64.3	1		1	
Finland	15	0.3	2.09	1.26–3.47	1.91	1.14–3.17
Other Nordic	22	0.5	1.56	1.00–2.42	1.36	0.87–2.12
Europe	225	5.0	1.60	1.40–1.84	1.50	1.30–1.72
Asia + Oceania	140	3.1	1.96	1.65–2.33	1.79	1.50–2.13
Middle East	240	5.3	2.00	1.75–2.28	1.88	1.64–2.16
Africa	198	4.4	4.89	4.23–5.65	4.39	3.77–5.10
North America	20	0.4	2.12	1.35–3.34	1.94	1.23–3.05
South America	64	1.4	2.13	1.66–2.73	2.12	1.65–2.72
Bipolar disorder without psychosis						
Swedish-born (reference)	5130	81.5	1		1	
Finland	21	0.3	1.73	1.13–2.66	1.56	1.01–2.41
Other Nordic	31	0.5	1.44	1.01–2.05	1.35	0.94–1.93
Europe	110	1.8	0.45	0.37–0.54	0.43	0.36–0.53
Asia + Oceania	79	1.3	0.63	0.50–0.79	0.60	0.47–0.75
Middle East	88	1.4	0.42	0.34–0.52	0.43	0.34–0.53
Africa	23	0.4	0.33	0.22–0.50	0.32	0.21–0.48
North America	26	0.4	1.68	1.14–2.47	1.69	1.15–2.50
South America	62	1.0	1.16	0.90–1.49	1.27	0.99–1.63
Unknown	1	0.0	3.73	0.52–26.46	3.27	0.46–23.27

95% CI, 95% confidence interval. There were no cases of schizophrenia/schizoaffective disorder, affective psychosis, or other non-affective psychosis among people of ‘unknown’ region of origin.

a Adjusted for age, sex and time period.

Risks of all psychotic disorders were elevated across most ages-of-migration compared with the Swedish-born population (Table 4). For schizophrenia and schizoaffective disorder elevated risk was apparent across all ages-of-migration relative to the Swedish-born population, and was highest for those migrating in adolescence and early adulthood (aHR_adolescence_: 2.38, 95% CI 1.79–3.16; aHR_early adulthood_: 2.77, 95% CI 1.64–4.69). For other non-affective psychotic disorders risk was elevated at all ages-of-migration, except early adulthood (aHR: 1.17; 95% CI 0.80–1.72). Risk of affective psychosis was elevated among those who migrated in infancy (aHR: 1.71, 95% CI 1.31–2.24), adolescence (aHR: 1.81, 95% CI 1.42–2.30) and early adulthood (aHR 2.05, 95% CI 1.20–3.52), but not childhood. In contrast, bipolar disorder without psychosis was associated with lower risks at all ages-of-migration, except during infancy (aHR: 1.20, 95% CI 1.01–1.42).

**Table 4.** Unadjusted and adjusted hazard ratios by Age-At-Migration

	*N*	%	Unadjusted		Adjusted	
			Hazard ratio	95% CI	Hazard ratio^a^	95% CI
Schizophrenia + schizoaffective						
Swedish born (reference)	1333	61.4	1		1	
Infancy (0–2)	49	2.3	1.57	1.17–2.09	1.57	1.18–2.09
Early childhood (3–6)	89	4.1	2.16	1.74–2.67	2.14	1.73–2.66
Middle childhood (7–12)	132	6.1	2.34	1.96–2.80	2.32	1.94–2.78
Adolescence (13–18)	105	4.8	2.97	2.43–3.62	2.76	2.23–3.41
Early adulthood (19–29)	96	4.4	3.56	2.88–4.41	2.77	1.64–4.69
Affective psychotic disorders						
** **Swedish born (reference)	1532	70.8	1		1	
** **Infancy (0–2)	58	2.7	1.69	1.30–2.21	1.71	1.31–2.24
** **Early childhood (3–6)	58	2.7	1.27	0.97–1.66	1.29	0.99–1.68
** **Middle childhood (7–12)	67	3.1	1.07	0.83–1.37	1.13	0.88–1.45
** **Adolescence (13–18)	87	4.0	1.89	1.51–2.38	1.81	1.42–2.30
** **Early adulthood (19–29)	102	4.7	2.34	1.90–2.89	2.05	1.20–3.52
Other non-affective psychotic disorders						
** **Swedish born (reference)	2899	64.3	1		1	
** **Infancy (0–2)	126	2.8	1.92	1.61–2.30	1.95	1.63–2.33
** **Early childhood (3–6)	191	4.2	2.16	1.86–2.50	2.15	1.85–2.49
** **Middle childhood (7–12)	210	4.7	1.76	1.52–2.02	1.82	1.58–2.10
** **Adolescence (13–18)	207	4.6	2.58	2.24–2.98	2.16	1.86–2.52
** **Early adulthood (19–29)	190	4.2	2.58	2.22–2.99	1.17	0.80–1.72
Bipolar disorder without psychosis						
Swedish born (reference)	5130	81.5	1		1	
Infancy (0–2)	141	2.2	1.19	1.00–1.40	1.20	1.01–1.42
Early childhood (3–6)	83	1.3	0.53	0.42–0.66	0.54	0.43–0.67
Middle childhood (7–12)	97	1.5	0.45	0.36–0.55	0.49	0.40–0.59
Adolescence (13–18)	43	0.7	0.31	0.23–0.42	0.27	0.20–0.37
Early adulthood (19–29)	77	1.2	0.68	0.55–0.86	0.35	0.20–0.61

95% CI, 95% confidence interval; Swedish-born reference category. Children of migrants excluded as results shown in Table 2.

a Adjusted for age, sex, and time period, baseline Swedish-born.

These associations were partially attenuated following adjustment for income, but this did not substantially alter our findings (online Supplementary Tables S1–S3). A sensitivity analysis excluding possible prevalent cases in migrants (diagnosed within 2 calendar years of immigration) did not change the pattern of our results (online Supplementary Tables S4–S6). Schoenfeld tests and examination of log-log residual plots revealed no evidence of departure from proportionality across our main exposures for schizophrenia or affective psychosis (online Supplementary Table S7). These tests did suggest a departure from proportionality for our other two outcomes, but log-log residual plots revealed these effects were small and the departure from a zero-slope was negligible (online Supplementary Fig. S1).

## Discussion

### Principal findings

This is the first longitudinal study to investigate how migrant status, region of origin, and age-at-migration affect the risk of schizophrenia, schizoaffective disorder, affective psychotic disorders, other non-affective psychotic disorders, and non-psychotic bipolar disorder. We discovered distinct signatures of risk, which varied according to the presence or absence of psychosis. Thus, migration-related exposures substantially increased the risk of psychotic disorders, albeit with more attenuated effect sizes for affective psychoses. In contrast, non-psychotic bipolar disorder showed a markedly different pattern, with generally lower risks across our three migration-related exposures compared with the Swedish-born population. Our results were impervious to adjustment for income and were unlikely to be explained by prevalent cases amongst migrants, or by age, sex, or period effects.

### Strengths and limitations

We used to register data from a nationwide cohort of nearly 1.8 million people, with nearly complete coverage, and virtually no loss-to-follow-up. Clinical recording of schizophrenia spectrum disorders is known to be highly complete in the registers with good validity (Dalman *et al*., 2002; Hollander *et al*., 2016), although this requires demonstrating for bipolar disorders. We limited our follow-up period to 1997–2011 when ICD-10 was the sole diagnostic classification system used in Sweden. This avoided major changes in diagnostic practice over the cohort period and allowed us to distinguish between bipolar disorders with and without psychosis for the first time in this population. Our decision to use a hierarchical diagnostic classification did not bias findings; similar results were obtained when we classified participants by their first or final diagnosis in the registers (data available from authors). To account for possible changes in case ascertainment over time, including the introduction of more outpatient services in Sweden after 1997, we included calendar time to control for period effects. There was no evidence of a violation of proportional hazards across our three migration-related exposures for schizophrenia or affective psychosis. Departures from proportionality for other outcomes were small, and were likely driven by our large sample size, and would likely have had trivial effects on the interpretation of our results.

We have no reason to believe that our estimates of the incidence of bipolar disorder and affective psychosis are not valid, however, unlike non-affective psychosis, these disorders still require validation in the Swedish registry data. In our sample, psychosis was only diagnosed in 25.6% of those with bipolar disorder/mania, which is lower than previous lifetime estimates between 50 and 61% (Goodwin and Jamison, 1990; Dunayevich and Keck, 2000). This may reflect the young age of our sample, meaning that some people diagnosed with bipolar disorder without psychosis for the first time in our study may eventually go onto experience an episode of psychosis. Alternatively, bipolar disorder with psychosis may be under-diagnosed in Sweden. Nonetheless, the overall incidence of affective psychosis in our sample (15.9, 95% CI 15.2–16.6) was higher than reported in previous studies (Kirkbride *et al*., 2006), favouring the former explanation than one of under-diagnosis. For all migration exposures, elevated risk of schizophrenia and schizoaffective disorders was of the order of magnitude higher than for affective psychosis. For children of migrants (although not migrants) the confidence intervals around the elevated risk of affective psychosis narrowly overlapped those for the non-significant difference in risk of bipolar disorder without psychosis between children of migrants and the Swedish-born populations; we cannot, therefore, exclude the possibility that our findings have partially arisen from chance, although the consistency and pattern of our results suggest this is unlikely; nevertheless, further, large studies are required to replicate our results.

In our study, case participants were based on contact with mental health services, which may have led to some underestimation of the true incidence of psychiatric disorders within the population, although register-based approaches appear to lead to more complete case ascertainment than first contact designs (Hogerzeil *et al*., 2014). Nevertheless, if migrants and their children had differential treatment-seeking behaviours compared with Swedish-born individuals, differential ascertainment bias could have affected our results. Research from the Netherlands has demonstrated a larger gap between mental health care need and service use among migrants compared with native Dutch (Koopmans *et al*., 2012). Despite the universal availability of health care in Sweden, such differences in help-seeking may have conservatively biased our estimates of risk amongst some migrants, particularly for less severe phenotypes such as bipolar disorder without psychosis for which people may be less likely to seek mental health care. Nevertheless, our findings for bipolar disorders without psychosis are in line with previous studies which have not consistently found elevated risk in migrants or their children (Swinnen and Selten, 2007; Cantor-Graae and Pedersen, 2013; Pignon *et al*., 2017).

Our sensitivity analyses suggested our results were not due to confounding by income. Nevertheless, our income covariate was differentially missing by migrant status (7.4% in migrants, 0.5% in their children, 0.1% in the Swedish-born), which may have introduced bias vis-à-vis residual confounding; from the available data, however, any confounding effect appeared modest, and income may lie on the causal pathway between migration and mental health, making adjustment inappropriate.

Further sensitivity analyses suggested our results were unlikely to be attributable to prevalent cases amongst migrants, arguing against selective migration, consistent with previous observations (Selten *et al*., 2002; van der Ven *et al*., 2014). Migration is a cognitively-demanding process, requiring effective planning skills, which would be inconsistent with premorbid cognitive defects experienced by people who go onto develop psychosis (Rosenthal *et al*., 1974; Cannon *et al*., 2000; van der Ven *et al*., 2014).

### Comparison with the previous literature

Our results are consistent with previous research demonstrating an increased risk of schizophrenia-spectrum disorders among migrants and their children (Lloyd *et al*., 2005; Coid *et al*., 2008; Cantor-Graae and Pedersen, 2013). Some studies have also shown an increased risk of affective psychosis in migrants and their children (Lloyd *et al*., 2005; Coid *et al*., 2008). Our results also mirror the wider literature, which has shown that region of origin has a significant impact on psychosis risk (Kirkbride *et al*., 2012*a*; Pedersen and Cantor-Graae, 2012), notably amongst visible minority populations (Fearon *et al*., 2006; Coid *et al*., 2008; Kirkbride *et al*., 2012*a*).

Comparisons with previous findings with respect to age-at-migration and psychotic disorders require careful attention. In one study (Veling *et al*., 2011), the highest risk of non-affective psychoses amongst migrants relative to the background population was associated with migration during infancy, with risk declining thereafter, such that adulthood migration was not associated with differences in risk. Two further studies (Anderson *et al*., 2015; Kirkbride *et al*., 2017*b*) found some evidence that elevated psychosis risk peaked with migration during adolescence relative to the background population, consistent with our observations for non-affective psychotic disorders. Nevertheless, in all these studies, including our own, overlapping confidence intervals for risks at different ages-of-migration mean these findings are also consistent with those reported by Cantor-Graae and Pedersen and Pedersen (2003) in Denmark, who found no statistically significant differences in psychosis risk by age-of-migration, when migration at birth provided the reference category. In our study, the only robust differences in risk by age-of-migration occurred for bipolar disorder without psychosis, never previously examined, where risks were elevated with infant migration, but significantly reduced at all other ages of migration, relative to the Swedish-born population.

### Meaning of findings

The different signatures of risk we observed between psychotic and non-psychotic disorders with respect to migration suggest that exposure to adversities related to migration and minority status could act specifically on psychotic rather than affective pathways. Emerging research supports the hypothesis that psychotic and non-psychotic bipolar disorder may have distinct neurodevelopmental origins (Murray *et al*., 2004; Maggioni *et al*., 2017). For example, structural magnetic resonance imaging has revealed that non-psychotic bipolar disorders do not show deficits in cortical grey matter volume, in contrast to affective psychosis and other psychotic disorders (Maggioni *et al*., 2017). Moreover, schizophrenia (Green, 2006) and bipolar disorder with psychosis (Glahn *et al*., 2007; Bora *et al*., 2010; Hill *et al*., 2013) also appear to share premorbid cognitive deficits, not consistently observed for non-psychotic affective disorders (Reichenberg *et al*., 2002; Trotta *et al*., 2014). These deficits could contribute to a plausible pathway, through which repeated exposure to social adversities, including migration, leads to psychotic symptoms via increased stress reactivity (Bentall *et al*., 2014). Individuals who develop non-psychotic bipolar disorder may not experience comparable premorbid cognitive deficits (Goodwin *et al*., 2008; MacCabe *et al*., 2010; Vreeker *et al*., 2016). However, the evidence remains equivocal, as a recent meta-analysis did not find different trajectories of cognitive functioning between schizophrenia and bipolar disorder (Bora and Özerdem, 2017), though did not differentiate between those with and without psychosis in the latter.

Repeated exposure to factors associated with migration – including social disadvantage, trauma or discrimination experienced prior to, during, or following migration – may also prime individuals to expect a greater level of threat from the environment (Reininghaus *et al*., 2016). Such processes may sensitize neurobiological pathways relevant to psychosis, including dysregulation of the mesolimbic dopaminergic system (Kapur, 2003), particularly amongst those with impaired neurocognition, who may be more prone to errors in salience appraisal of irrelevant or benign environmental stimuli (Kapur, 2003; Roiser *et al*., 2009). Given the absence of similar neurocognitive impairments in non-psychotic bipolar disorder (Kapur, 2003; Roiser *et al*., 2009), such psychological and biological mechanisms may partially underlie the observed differences between psychotic and non-psychotic disorders.

We should also give credence to another possible explanation of our results: misdiagnosis. It has been suggested that higher rates of psychotic disorders in some ethnic minority groups are due to diagnostic bias, with people from the majority group less likely to receive a stigmatizing diagnosis such as schizophrenia compared with bipolar disorder (Schwartz and Blankenship, 2014). Although we did not have data on ethnicity in our study, migrants from regions where they were most likely to be visible minorities in Sweden (i.e. Africa, the Middle East, Asia & Oceania) were more likely to be diagnosed with a psychotic disorder, and less likely to be diagnosed with bipolar disorder without psychosis than Swedish-born participants. Together with the finding that only migrants from Nordic countries or North America were more likely to be diagnosed with the non-psychotic bipolar disorder than the Swedish-born population, this is consistent with the misdiagnosis hypothesis. Nonetheless, our data do not entirely support this explanation. Finnish, other Nordic and European, and North America migrants were also all at increased risk of psychotic disorders, to a similar extent to groups from the Middle East, Asia, and South America. Furthermore, in our data, European migrants were also less likely to be diagnosed with the non-psychotic bipolar disorder, which does not readily fit with the hypothesis that people of white ethnicities are likely to receive less stigmatizing (non-psychotic) diagnoses instead of psychotic ones. In general, the misdiagnosis hypothesis is not borne out by other available evidence (Lewis *et al*., 1990; Hickling *et al*., 1999; Selten *et al*., 2010; Zandi *et al*., 2010), and previous epidemiological studies – with some blinding of diagnosticians to ethnicity – have demonstrated elevated rates of psychotic disorders persist in several black and minority ethnic groups (Fearon *et al*., 2006).

## Conclusion

The shared patterns of risk across three categories of psychotic disorders with respect to various migration-related exposures suggest that migration may act specifically on psychotic rather than affective pathways to disorder. This provides potentially important clues to the aetiology of serious mental illnesses and should galvanise efforts to identify the exact social, environmental, and biological determinants of this preventable, gross inequality experienced by migrant and minority populations (Kirkbride, 2017).

## Supplementary material

For supplementary material accompanying this paper visit https://doi.org/10.1017/S0033291718003227. click here to view supplementary material

## References

[ref1] Anderson KK, Cheng J, Susser E, McKenzie KJ and Kurdyak P (2015) Incidence of psychotic disorders among first-generation immigrants and refugees in Ontario. Canadian Medical Association Journal 187, E279–E286. 25964387 10.1503/cmaj.141420 PMC4467956

[ref2] Bentall RP, De Sousa P, Varese F, Wickham S, Sitko K, Haarmans M and Read J (2014) From adversity to psychosis: pathways and mechanisms from specific adversities to specific symptoms. Social Psychiatry and Psychiatric Epidemiology 49, 1011–1022. 24919446 10.1007/s00127-014-0914-0

[ref3] Björkenstam E, Björkenstam C, Hjern A, Reutfors J and Bodén R (2013) A five year diagnostic follow-up of 1840 patients after a first episode non-schizophrenia and non-affective psychosis. Schizophrenia Research 150, 205–210. 23899998 10.1016/j.schres.2013.07.011

[ref4] Bora E and Özerdem A (2017) Meta-analysis of longitudinal studies of cognition in bipolar disorder: comparison with healthy controls and schizophrenia. Psychological Medicine 47, 2753–2766. 28585513 10.1017/S0033291717001490

[ref5] Bora E, Yücel M and Pantelis C (2010) Cognitive impairment in affective psychoses: a meta-analysis. Schizophrenia Bulletin 36, 112–125. 19767349 10.1093/schbul/sbp093 PMC2800140

[ref6] Bourque F, van der Ven E and Malla A (2011) A meta-analysis of the risk for psychotic disorders among first- and second-generation immigrants. Psychological Medicine 41, 897–910. 20663257 10.1017/S0033291710001406

[ref7] Cannon TD, Bearden CE, Hollister JM, Rosso IM, Sanchez LE and Hadley T (2000) Childhood cognitive functioning in schizophrenia patients and their unaffected siblings: a prospective cohort study. Schizophrenia Bulletin 26, 379–393. 10885638 10.1093/oxfordjournals.schbul.a033460

[ref8] Cantor-Graae E and Pedersen CB (2013) Full spectrum of psychiatric disorders related to foreign migration. JAMA Psychiatry 70, 427. 23446644 10.1001/jamapsychiatry.2013.441

[ref9] Cantor-Graae E and Selten J-PP (2005) Schizophrenia and migration: a meta-analysis and review. American Journal of Psychiatry 162, 12–24. 15625195 10.1176/appi.ajp.162.1.12

[ref10] Close C, Kouvonen A, Bosqui T, Patel K, O'Reilly D and Donnelly M (2016) The mental health and wellbeing of first generation migrants: a systematic-narrative review of reviews. Globalization and Health 12, 47. 27558472 10.1186/s12992-016-0187-3 PMC4997738

[ref11] Coid JW, Kirkbride JB, Barker D, Cowden F, Stamps R, Yang M and Jones PB (2008) Raised incidence rates of all psychoses among migrant groups: findings from the East London first episode psychosis study. Archives of General Psychiatry 65, 1250–1258. 18981336 10.1001/archpsyc.65.11.1250

[ref12] Colvert E, Rutter M, Kreppner J, Beckett C, Castle J, Groothues C, Hawkins A, Stevens S and Sonuga-Barke EJS (2008) Do theory of mind and executive function deficits underlie the adverse outcomes associated with profound early deprivation?: Findings from the English and Romanian Adoptees Study. Journal of Abnormal Child Psychology 36, 1057–1068. 18427975 10.1007/s10802-008-9232-x

[ref13] Dalman C, Broms J, Cullberg J and Allebeck P (2002) Young cases of schizophrenia identified in a national inpatient register. Social Psychiatry and Psychiatric Epidemiology 37, 527–531. 12395142 10.1007/s00127-002-0582-3

[ref14] Dunayevich E and Keck PE (2000) Prevalence and description of psychotic features in bipolar mania. Current Psychiatry Reports 2, 286–290. 11122970 10.1007/s11920-000-0069-4

[ref15] Fearon P and Morgan C (2006) Environmental factors in schizophrenia: the role of migrant studies. Schizophrenia Bulletin 32, 405–408. 16699062 10.1093/schbul/sbj076 PMC2632238

[ref16] Fearon P, Kirkbride JB, Morgan C, Dazzan P, Morgan K, Lloyd T, Hutchinson G, Tarrant J, Fung WLA, Holloway J, Mallett R, Harrison G, Leff J, Jones PB and Murray RM (2006) Incidence of schizophrenia and other psychoses in ethnic minority groups: results from the MRC AESOP Study. Psychological Medicine 36, 1541–1550. 16938150 10.1017/S0033291706008774

[ref17] Garety P, Kuipers E, Fowler D, Freeman D and Bebbington P (2001) A cognitive model of the positive symptoms of psychosis. Psychological Medicine 31, 189–195. 11232907 10.1017/s0033291701003312

[ref18] Glahn DC, Bearden CE, Barguil M, Barrett J, Reichenberg A, Bowden CL, Soares JC and Velligan DI (2007) The neurocognitive signature of psychotic bipolar disorder. Biological Psychiatry 62, 910–916. 17543288 10.1016/j.biopsych.2007.02.001

[ref19] Goodwin F and Jamison K (1990) Manic Depressive Illness. New York: Oxford University Press.

[ref20] Goodwin GM, Martinez-Aran A, Glahn DC and Vieta E (2008) Cognitive impairment in bipolar disorder: neurodevelopment or neurodegeneration? An ECNP expert meeting report. European Neuropsychopharmacology 18, 787–793. 18725178 10.1016/j.euroneuro.2008.07.005

[ref21] Green MF (2006) Cognitive impairment and functional outcome in schizophrenia and bipolar disorder. Journal of Clinical Psychiatry 67, 3–8. 16965182

[ref22] Hickling FW, Mckenzie K, Mullen R and Murray R (1999) A Jamaican psychiatrist evaluates diagnoses at a London psychiatric hospital. British Journal of Psychiatry 175, 283–285. 10645332 10.1192/bjp.175.3.283

[ref23] Hill S, Reilly J, Keefe R, Gold J, Bishop J, Gershon E, Tamminga C, Pearlson G, Keshavan M and Sweeney J (2013) Neuropsychological impairments in schizophrenia and psychotic bipolar disorder: findings from the Bipolar and Schizophrenia Network on Intermediate Phenotypes (B-SNIP) Study. The American Journal of Psychiatry 170, 1275–1284. 23771174 10.1176/appi.ajp.2013.12101298 PMC5314430

[ref24] Hogerzeil SJ, van Hemert AM, Rosendaal FR, Susser E and Hoek HW (2014) Direct comparison of first-contact versus longitudinal register-based case finding in the same population: early evidence that the incidence of schizophrenia may be three times higher than commonly reported. Psychological Medicine 44, 3481–3490. 25066605 10.1017/S003329171400083X

[ref25] Hollander A-C, Dal H, Lewis G, Magnusson C, Kirkbride JB and Dalman C (2016) Refugee migration and risk of schizophrenia and other non-affective psychoses: cohort study of 1.3 million people in Sweden. BMJ 352, 1–8. 10.1136/bmj.i1030 PMC4793153 26979256

[ref26] Kapur S (2003) Psychosis as a state of aberrant salience: a framework linking biology, phenomenology, and pharmacology in schizophrenia. American Journal of Psychiatry 160, 13–23. 12505794 10.1176/appi.ajp.160.1.13

[ref27] Kirkbride JB (2017) Migration and psychosis: our smoking lung? World Psychiatry 16, 119–120. 28498570 10.1002/wps.20406 PMC5428174

[ref28] Kirkbride JB, Fearon P, Morgan C, Dazzan P, Morgan K, Tarrant J, Lloyd T, Holloway J, Hutchinson G, Leff JP, Mallett RM, Harrison GL, Murray RM and Jones PB (2006) Heterogeneity in incidence rates of schizophrenia and other psychotic syndromes: findings from 3-center AESOP study. Archives of General Psychiatry 63, 250–258. 16520429 10.1001/archpsyc.63.3.250

[ref29] Kirkbride JB, Barker D, Cowden F, Stamps R, Yang M, Jones PB and Coid JW (2008) Psychoses, ethnicity and socio-economic status. British Journal of Psychiatry 193, 18–24. 18700213 10.1192/bjp.bp.107.041566

[ref30] Kirkbride JB, Errazuriz A, Croudace T, Morgan C, Jackson D, McCrone P, Murray R and Jones P (2012 *a*) Systematic Review of the Incidence and Prevalence of Schizophrenia and Other Psychoses in England. London: Department of Health Policy Research Programme.

[ref31] Kirkbride JB, Errazuriz A, Croudace TJ, Morgan C, Jackson D, Boydell J, Murray RM and Jones PB (2012 *b*) Incidence of schizophrenia and other psychoses in England, 1950-2009: a systematic review and meta-analyses. PloS ONE 7, e31660. 22457710 10.1371/journal.pone.0031660 PMC3310436

[ref32] Kirkbride JB, Hameed Y, Ankireddypalli G, Ioannidis K, Crane CM, Nasir M, Kabacs N, Metastasio A, Jenkins O, Espandian A, Spyridi S, Ralevic D, Siddabattuni S, Walden B, Adeoye A, Perez J and Jones PB (2017 *a*) The epidemiology of first-episode psychosis in early intervention in psychosis services: Findings From the Social Epidemiology of Psychoses in East Anglia [SEPEA] Study. American Journal of Psychiatry 147, 143–153. 10.1176/appi.ajp.2016.16010103 PMC5939990 27771972

[ref33] Kirkbride JB, Hameed Y, Ioannidis K, Ankireddypalli G, Crane CM, Nasir M, Kabacs N, Metastasio A, Jenkins O, Espandian A, Spyridi S, Ralevic D, Siddabattuni S, Walden B, Adeoye A, Perez J and Jones PB (2017 *b*) Ethnic minority status, age-at-immigration and psychosis risk in rural environments: evidence from the SEPEA Study. Schizophrenia Bulletin 43, 1251–1261. 28521056 10.1093/schbul/sbx010 PMC5737276

[ref34] Koopmans GT, Uiters E, Devillé W and Foets M (2012) The use of outpatient mental health care services of migrants vis-à-vis Dutch natives: equal access? International Journal of Social Psychiatry 59, 342–350. 22392446 10.1177/0020764012437129

[ref35] Lewis G, Croft-Jeffreys C and David A (1990) Are British psychiatrists racist? British Journal of Psychiatry 157, 410–415. 2245273 10.1192/bjp.157.3.410

[ref36] Lloyd T, Kennedy N, Fearon P, Kirkbride J, Mallett R, Leff J, Holloway J, Harrison G, Dazzan P, Morgan K, Murray RM and Jones PB (2005) Incidence of bipolar affective disorder in three UK cities: results from the ÆSOP study. British Journal of Psychiatry 186, 126–131. 15684235 10.1192/bjp.186.2.126

[ref37] MacCabe JH, Lambe MP, Cnattingius S, Sham PC, David AS, Reichenberg A, Murray RM and Hultman CM (2010) Excellent school performance at age 16 and risk of adult bipolar disorder: national cohort study. British Journal of Psychiatry 196, 109–115. 20118454 10.1192/bjp.bp.108.060368

[ref38] Maggioni E, Altamura AC and Brambilla P (2017) Exploring the neuroanatomical bases of psychotic features in bipolar disorder. Epidemiology and Psychiatric Sciences 26, 358–363. 28343462 10.1017/S2045796017000087 PMC6998627

[ref39] Murray RM, Sham P, Van Os J, Zanelli J, Cannon M and McDonald C (2004) A developmental model for similarities and dissimilarities between schizophrenia and bipolar disorder. Schizophrenia Research 71, 405–416. 15474912 10.1016/j.schres.2004.03.002

[ref40] Ødegaard Ø (1932) Emigration and insanity. Acta Psychiatrica Neurologica (Supp.), 4, 1–206.

[ref41] OECD (2017) Foreign-born employment (indicator). doi: 10.1787/05428726-en (Accessed on 12 November 2017).

[ref42] Patel V and Goodman A (2007) Researching protective and promotive factors in mental health. International Journal of Epidemiology 36, 703–707. 17646185 10.1093/ije/dym147

[ref43] Pedersen CB and Cantor-Graae E (2012) Age at migration and risk of schizophrenia among immigrants in Denmark: a 25-year incidence study. The American Journal of Psychiatry 169, 1117–1118. 23032390 10.1176/appi.ajp.2012.12050614

[ref44] Perner J and Lang B (1999) Development of theory of mind and executive control. Trends in Cognitive Science 3, 337–344. 10.1016/s1364-6613(99)01362-5 10461196

[ref45] Pignon B, Alexis Geoffroy P, Thomas P, Roelandt JL, Rolland B, Morgan C, Vaiva G and Amad A (2017) Prevalence and clinical severity of mood disorders among first-, second- and third-generation migrants. Journal of Affective Disorders 210, 174–180. 28049102 10.1016/j.jad.2016.12.039

[ref46] Reichenberg A, Weiser M, Rabinowitz J, Caspi A, Schmeidler J, Mark M, Kaplan Z and Davidson M (2002) A population-based cohort study of premorbid intellectual, language, and behavioral functioning in patients with schizophrenia, schizoaffective disorder, and nonpsychotic bipolar disorder. Psychiatry: Interpersonal and Biological Processes 159, 2027–2035. 10.1176/appi.ajp.159.12.2027 12450952

[ref47] Reininghaus U, Kempton MJ, Valmaggia L, Craig TKJ, Garety P, Onyejiaka A, Gayer-Anderson C, So SH, Hubbard K, Beards S, Dazzan P, Pariante C, Mondelli V, Fisher HL, Mills JG, Viechtbauer W, McGuire P, van Os J, Murray RM, Wykes T, Myin-Germeys I and Morgan C (2016) Stress sensitivity, aberrant salience, and threat anticipation in early psychosis: an experience sampling study. Schizophrenia Bulletin 42, sbv190. 10.1093/schbul/sbv190 PMC4838104 26834027

[ref48] Roiser JP, Stephan KE, den Ouden HEM, Barnes TRE, Friston KJ and Joyce EM (2009) Do patients with schizophrenia exhibit aberrant salience? Psychological medicine 39, 199–209. 18588739 10.1017/S0033291708003863 PMC2635536

[ref49] Rosenthal D, Goldberg I, Jacobsen B, Wender P, Kety S, Schulsinger F and Eldred C (1974) Migration, heredity, and schizophrenia. Psychiatry 37, 321–339. 4427966 10.1080/00332747.1974.11023818

[ref50] Rothmond DA, Weickert CS and Webster MJ (2012) Developmental changes in human dopamine neurotransmission: cortical receptors and terminators. BMC Neuroscience 13, 18. 22336227 10.1186/1471-2202-13-18 PMC3315415

[ref51] Schwartz RC and Blankenship DM (2014) Racial disparities in psychotic disorder diagnosis: a review of empirical literature. World Journal of Psychiatry 4, 133–140. 25540728 10.5498/wjp.v4.i4.133 PMC4274585

[ref52] Selemon LD and Zecevic N (2015) Schizophrenia: a tale of two critical periods for prefrontal cortical development. Translational Psychiatry 5, 1–11. 10.1038/tp.2015.115 PMC4564568 26285133

[ref53] Selten JP, Cantor-Graae E, Slaets J and Kahn RS (2002) Ødegaard's selection hypothesis revisited: schizophrenia in Surinamese immigrants to the Netherlands. American Journal of Psychiatry 159, 669–671. 11925311 10.1176/appi.ajp.159.4.669

[ref54] Selten JP, Laan W, Veen ND, Blom JD, Veling W and Hoek HW (2010) Incidence of schizophrenia among Moroccan immigrants to the Netherlands. Schizophrenia Research 124, 240–241. 20813502 10.1016/j.schres.2010.08.010

[ref55] Swinnen S and Selten J-P (2007) Mood disorders and migration. British Journal of Psychiatry 190, 6–10. 17197650 10.1192/bjp.bp.105.020800

[ref56] Trotta A, Murray RM and MacCabe JH (2014) Do premorbid and post-onset cognitive functioning differ between schizophrenia and bipolar disorder? A systematic review and meta-analysis. Psychological Medicine 45, 381–394. 25065268 10.1017/S0033291714001512

[ref57] van der Ven E, Dalman C, Wicks S, Allebeck P, Magnusson C, van Os J and Selten JP (2015) Testing Ødegaard's selective migration hypothesis: a longitudinal cohort study of risk factors for non-affective psychotic disorders among prospective emigrants. Psychological Medicine 45, 727–34. 25084213 10.1017/S0033291714001780

[ref58] Veling W, Hoek HW, Selten J-P and Susser E (2011) Age at migration and future risk of psychotic disorders among immigrants in the Netherlands: a 7-year incidence study. The American Journal of Psychiatry 168, 1278–1285. 22193672 10.1176/appi.ajp.2011.11010110

[ref59] Vreeker A, Boks MPM, Abramovic L, Verkooijen S, van Bergen AH, Hillegers MHJ, Spijker AT, Hoencamp E, Regeer EJ, Riemersma-Van der Lek RF, Stevens AWMM, Schulte PFJ, Vonk R, Hoekstra R, van Beveren NJM, Kupka RW, Brouwer RM, Bearden CE, MacCabe JH and Ophoff RA (2016) High educational performance is a distinctive feature of bipolar disorder: a study on cognition in bipolar disorder, schizophrenia patients, relatives and controls. Psychological Medicine 46, 807–818. 26621616 10.1017/S0033291715002299 PMC5824688

[ref60] Zandi T, Havenaar JM, Smits M, Limburg-Okken AG, van Es H, Cahn W, Algra A, Kahn RS and van den Brink W (2010) First contact incidence of psychotic disorders among native Dutch and Moroccan immigrants in the Netherlands: influence of diagnostic bias. Schizophrenia Research 119, 27–33. 20332065 10.1016/j.schres.2010.02.1059

