# Supplementary material for: Risk of schizophrenia, schizoaffective, and bipolar disorders by migrant status, region of origin, and age-at-migration: a national cohort study of 1.8 million people
Source: Psychol Med. 2018 Dec 5;49(14):2354–63. doi: 10.1017/S0033291718003227 (PMC6763532; doi:10.1017/S0033291718003227)
Supplement: Supplementary file 1 [file S0033291718003227sup001.docx]

**Online Supplement: Risk of schizophrenia, schizoaffective, and bipolar disorders by migrant status, region of origin, and age-at-migration. A national cohort study of 1.8 million people**

## Supplemental table 1: Income-Adjusted hazard ratios by migrant status

|  |  |  | Adjusted^1^ | | |
| --- | --- | --- | --- | --- | --- |
|  | N | % | Hazard ratio | 95%CI | |
| **Schizophrenia + schizoaffective** |  |  |  |  | |
| Swedish born (reference) | 1,333 | 61.4% | 1 |  |  |
| Children of migrants | 368 | 16.9% | **1.84** | **1.63** | **2.06** |
| Migrant | 471 | 21.7% | **1.83** | **1.62** | **2.07** |
| **Affective psychotic disorders** |  |  |  |  |  |
| Swedish born (reference) | 1,532 | 70.8% | 1 |  |  |
| Children of migrants | 259 | 12.0% | **1.17** | **1.03** | **1.34** |
| Migrant | 372 | 17.2% | **1.29** | **1.12** | **1.49** |
| **Other non-affective psychotic disorders** |  |  |  |  |  |
| Swedish born (reference) | 2,899 | 64.3% | 1 |  |  |
| Children of migrants | 687 | 15.2% | **1.54** | **1.42** | **1.68** |
| Migrant | 924 | 20.5% | **1.64** | **1.50** | **1.80** |
| **Bipolar disorder without psychosis** |  |  |  |  |  |
| Swedish born (reference) | 5,130 | 81.5% | 1 |  |  |
| Children of migrants | 724 | 11.5% | 0.94 | 0.87 | 1.01 |
| Migrant | 441 | 7.0% | **0.52** | **0.47** | **0.58** |

HR: Hazard ratio; 95%CI: 95% confidence interval

^1^Adjusted for age, sex, time period, and income

**Supplemental Table 2: income-adjusted hazard ratios by region of origin**

|  | N | % | Adjusted HR^1^ | 95%CI | |
| --- | --- | --- | --- | --- | --- |
| **Schizophrenia + schizoaffective** |  |  |  |  |  |
| Sweden | 1,333 | 61.4% | 1 |  |  |
| Finland | 7 | 0.3% | 1.48 | 0.66 | 3.31 |
| Other Nordic | 10 | 0.5% | 1.38 | 0.74 | 2.59 |
| Europe | 125 | 5.8% | **1.55** | **1.28** | **1.89** |
| Asia + Oceania | 61 | 2.8% | **1.57** | **1.20** | **2.06** |
| Middle East | 123 | 5.7% | **1.62** | **1.33** | **1.98** |
| Africa | 105 | 4.8% | **4.32** | **3.46** | **5.38** |
| North America | 11 | 0.5% | **1.99** | **1.06** | **3.71** |
| South America | 29 | 1.3% | **1.74** | **1.18** | **2.54** |
| **Affective psychotic disorders** |  |  |  |  |  |
|  |  |  |  |  |  |
| Sweden | 1,532 | 70.8% | 1 |  |  |
| Finland | 6 | 0.3% | 1.39 | 0.62 | 3.12 |
| Other Nordic | 7 | 0.3% | 0.93 | 0.44 | 1.97 |
| Europe | 106 | 4.9% | 1.15 | 0.92 | 1.44 |
| Asia + Oceania | 71 | 3.3% | **1.40** | **1.06** | **1.85** |
| Middle East | 97 | 4.5% | 1.15 | 0.91 | 1.46 |
| Africa | 50 | 2.3% | **1.75** | **1.26** | **2.42** |
| North America | 7 | 0.3% | 1.36 | 0.65 | 2.88 |
| South America | 28 | 1.3% | **1.81** | **1.23** | **2.65** |
| **Other non-affective psychotic disorders** |  |  |  |  |  |
| Sweden | 2,899 | 64.3% | 1 |  |  |
| Finland | 15 | 0.3% | 1.61 | 0.95 | 2.73 |
| Other Nordic | 22 | 0.5% | 1.05 | 0.65 | 1.71 |
| Europe | 225 | 5.0% | **1.28** | **1.10** | **1.47** |
| Asia + Oceania | 140 | 3.1% | **1.56** | **1.29** | **1.87** |
| Middle East | 240 | 5.3% | **1.52** | **1.31** | **1.75** |
| Africa | 198 | 4.4% | **3.55** | **3.02** | **4.18** |
| North America | 20 | 0.4% | 1.46 | 0.89 | 2.40 |
| South America | 64 | 1.4% | **2.00** | **1.56** | **2.57** |
| **Bipolar disorder without psychosis** |  |  |  |  |  |
| Sweden | 5,130 | 81.5% | 1 |  |  |
| Finland | 21 | 0.3% | 1.42 | 0.90 | 2.24 |
| Other Nordic | 31 | 0.5% | 1.18 | 0.81 | 1.74 |
| Europe | 110 | 1.8% | **0.37** | **0.30** | **0.46** |
| Asia + Oceania | 79 | 1.3% | **0.58** | **0.46** | **0.74** |
| Middle East | 88 | 1.4% | **0.38** | **0.30** | **0.47** |
| Africa | 23 | 0.4% | **0.29** | **0.19** | **0.44** |
| North America | 26 | 0.4% | **1.58** | **1.06** | **2.34** |
| South America | 62 | 1.0% | 1.22 | 0.95 | 1.58 |
| Unknown | 1 | 0.0% | 3.20 | 0.45 | 22.78 |

**Bold** denotes p<0.05

HR: Hazard ratio; 95%CI: 95% confidence interval

^1^Adjusted for age, sex, time period, and income

**Supplemental table 3: Income-adjusted hazard ratios by Age-At-Migration**

|  | N | % | Adjusted HR^1^ | 95%CI |  |
| --- | --- | --- | --- | --- | --- |
| **Schizophrenia + schizoaffective** |  |  |  |  |  |
| Swedish born (reference) | 1,333 | 61.4% | 1 |  |  |
| Infancy (0-2) | 49 | 2.3% | **1.54** | **1.15** | **1.15** |
| Early childhood (3-6) | 89 | 4.1% | **1.82** | **1.46** | **1.46** |
| Middle childhood (7-12) | 132 | 6.1% | **1.97** | **1.64** | **1.64** |
| Adolescence (13-18) | 105 | 4.8% | **1.84** | **1.45** | **1.45** |
| Early adulthood (19-29) | 96 | 4.4% | **1.98** | **1.15** | **1.15** |
| **Affective psychotic disorders** |  |  |  |  |  |
| Swedish born (reference) | 1,532 | 70.8% | 1 |  |  |
| Infancy (0-2) | 58 | 2.7% | **1.70** | **1.30** | **1.30** |
| Early childhood (3-6) | 58 | 2.7% | 1.23 | 0.94 | 0.94 |
| Middle childhood (7-12) | 67 | 3.1% | 1.03 | 0.79 | 0.79 |
| Adolescence (13-18) | 87 | 4.0% | **1.40** | **1.06** | **1.06** |
| Early adulthood (19-29) | 102 | 4.7% | 1.41 | 0.78 | 0.78 |
| **Other non-affective psychotic disorders** |  |  |  |  |  |
| Swedish born (reference) | 2,899 | 64.3% | 1 |  |  |
| Infancy (0-2) | 126 | 2.8% | **1.87** | **1.56** | **1.56** |
| Early childhood (3-6) | 191 | 4.2% | **1.89** | **1.63** | **1.63** |
| Middle childhood (7-12) | 210 | 4.7% | **1.54** | **1.33** | **1.33** |
| Adolescence (13-18) | 207 | 4.6% | **1.39** | **1.17** | **1.17** |
| Early adulthood (19-29) | 190 | 4.2% | 0.74 | 0.50 | 0.50 |
| **Bipolar disorder without psychosis** |  |  |  |  |  |
| Swedish born (reference) | 5,130 | 81.5% | 1 |  |  |
| Infancy (0-2) | 141 | 2.2% | 1.17 | 0.99 | 0.99 |
| Early childhood (3-6) | 83 | 1.3% | **0.49** | **0.39** | **0.39** |
| Middle childhood (7-12) | 97 | 1.5% | **0.42** | **0.34** | **0.34** |
| Adolescence (13-18) | 43 | 0.7% | **0.20** | **0.14** | **0.14** |
| Early adulthood (19-29) | 77 | 1.2% | **0.27** | **0.15** | **0.15** |

**Bold** denotes p<0.05

HR: Hazard ratio; 95%CI: 95% confidence interval

^1^Adjusted for age, sex, time period, and income

**Supplemental table 4: Unadjusted and Adjusted hazard ratios by migrant status, Sensitivity analysis with washout period**

|  |  | Unadjusted | | |  | Adjusted^1^ | | |  |
| --- | --- | --- | --- | --- | --- | --- | --- | --- | --- |
|  | N | HR | 95%CI | |  | HR | | 95%CI |  |
| **Schizophrenia + schizoaffective** |  |  |  | |  |  | | |  |
| Swedish born (reference) | 1,333 | 63.7% | 1 |  |  | 1 |  | |  |
| Child of migrant(s) | 368 | 13.6% | **2.01** | **1.79** | **2.26** | **2.01** | **1.79** | | **2.26** |
| Migrant | 392 | 18.7% | **2.06** | **1.84** | **2.31** | **2.13** | **1.89** | | **2.40** |
| **Affective psychotic disorders** |  |  |  |  |  |  |  | |  |
| Swedish born (reference) | 1,532 | 73.2% | 1 |  |  | 1 |  | |  |
| Child of migrant(s) | 259 | 12.4% | **1.25** | **1.09** | **1.43** | **1.23** | **1.07** | | **1.40** |
| Migrant | 302 | 14.4% | **1.35** | **1.19** | **1.53** | **1.37** | **1.19** | | **1.57** |
| **Other non-affective psychotic disorders** |  |  |  |  |  |  |  | |  |
| Swedish born (reference) | 2,899 | 66.6% | 1 |  |  | 1 |  | |  |
| Child of migrant(s) | 687 | 15.8% | **1.70** | **1.56** | **1.85** | **1.69** | **1.55** | | **1.83** |
| Migrant | 768 | 17.6% | **1.81** | **1.67** | **1.96** | **1.93** | **1.77** | | **2.10** |
| **Bipolar disorder without psychosis** |  |  |  |  |  |  |  | |  |
| Swedish born (reference) | 5,130 | 82.1% | 1 |  |  | 1 |  | |  |
| Child of migrant(s) | 724 | 11.6% | 1.02 | 0.95 | 1.11 | 1.00 | 0.93 | | 1.09 |
| Migrant | 394 | 6.3% | **0.53** | **0.48** | **0.58** | **0.58** | **0.52** | | **0.65** |

**Bold** denotes p<0.05

HR: Hazard ratio; 95%CI: 95% confidence interval

^1^Adjusted for age, sex, and time period

**Supplemental table 5: Unadjusted and adjusted hazard ratios by region of origin, Sensitivity analysis with washout period**

|  | N | % | Unadjusted | | | Adjusted | | |
| --- | --- | --- | --- | --- | --- | --- | --- | --- |
|  |  |  | HR | 95% CI | | HR | 95% CI | |
| **Schizophrenia + schizoaffective** | |  |  |  | |  |  | |
| Sweden | 1,333 | 63.7% | 1 |  |  |  |  |  |
| Finland | 6 | 0.3% | 2.01 | 0.90 | 4.48 | 2.16 | 0.97 | 4.82 |
| Other Nordic | 8 | 0.4% | 1.53 | 0.76 | 3.06 | 1.59 | 0.79 | 3.20 |
| Europe | 108 | 5.2% | **1.76** | **1.45** | **2.14** | **1.83** | **1.50** | **2.23** |
| Asia + Oceania | 50 | 2.4% | **1.64** | **1.24** | **2.18** | **1.75** | **1.31** | **2.32** |
| Middle East | 104 | 5.0% | **1.92** | **1.57** | **2.35** | **1.99** | **1.62** | **2.43** |
| Africa | 81 | 3.9% | **4.71** | **3.76** | **5.90** | **4.93** | **3.92** | **6.20** |
| North America | 7 | 0.3% | 1.81 | 0.86 | 3.80 | 1.86 | 0.88 | 3.91 |
| South America | 28 | 1.3% | **1.87** | **1.27** | **2.75** | **1.87** | **1.27** | **2.76** |
| **Affective psychotic disorders** | |  |  |  |  |  |  |  |
| Sweden | 2,122 | 73.2% | 1 |  |  | 1 |  |  |
| Finland | 6 | 0.3% | 1.61 | 0.72 | 3.59 | 1.64 | 0.73 | 3.68 |
| Other Nordic | 7 | 0.3% | 1.04 | 0.50 | 2.20 | 1.09 | 0.52 | 2.30 |
| Europe | 87 | 4.2% | 1.22 | 0.99 | 1.52 | 1.22 | 0.98 | 1.53 |
| Asia + Oceania | 57 | 2.7% | **1.54** | **1.18** | **2.01** | **1.54** | **1.17** | **2.03** |
| Middle East | 78 | 3.7% | 1.24 | 0.99 | 1.56 | 1.26 | 0.99 | 1.60 |
| Africa | 34 | 1.6% | **1.58** | **1.12** | **2.23** | **1.59** | **1.12** | **2.27** |
| North America | 5 | 0.2% | 1.08 | 0.45 | 2.60 | 1.12 | 0.46 | 2.70 |
| South America | 28 | 1.3% | **1.79** | **1.22** | **2.62** | **1.89** | **1.29** | **2.77** |
| **Other non-affective psychotic disorders** |  |  |  |  |  |  |  |  |
| Sweden | 2,899 | 66.6% | 1 |  |  | 1 |  |  |
| Finland | 13 | 0.3% | **1.87** | **1.09** | **3.23** | **2.15** | **1.24** | **3.71** |
| Other Nordic | 16 | 0.4% | 1.21 | 0.73 | 2.02 | 1.33 | 0.80 | 2.22 |
| Europe | 197 | 4.5% | **1.42** | **1.23** | **1.64** | **1.50** | **1.30** | **1.74** |
| Asia + Oceania | 112 | 2.6% | **1.61** | **1.33** | **1.95** | **1.77** | **1.46** | **2.15** |
| Middle East | 198 | 4.6% | **1.68** | **1.45** | **1.94** | **1.79** | **1.54** | **2.07** |
| Africa | 154 | 3.5% | **3.89** | **3.31** | **4.58** | **4.22** | **3.57** | **4.99** |
| North America | 16 | 0.4% | **1.71** | **1.03** | **2.85** | **1.84** | **1.11** | **3.06** |
| South America | 62 | 1.4% | **2.06** | **1.60** | **2.66** | **2.18** | **1.69** | **2.81** |
| **Bipolar disorder without psychosis** | |  |  |  |  |  |  |  |
| Sweden | 5,130 | 82.1% | 1 |  |  | 1 |  |  |
| Finland | 16 | 0.3% | 1.33 | 0.81 | 2.17 | 1.47 | 0.90 | 2.41 |
| Other Nordic | 24 | 0.4% | 1.13 | 0.75 | 1.68 | 1.28 | 0.85 | 1.91 |
| Europe | 99 | 1.6% | **0.41** | **0.33** | **0.50** | **0.44** | **0.36** | **0.53** |
| Asia + Oceania | 77 | 1.2% | **0.62** | **0.49** | **0.78** | **0.67** | **0.53** | **0.85** |
| Middle East | 82 | 1.3% | **0.39** | **0.32** | **0.49** | **0.44** | **0.35** | **0.55** |
| Africa | 19 | 0.3% | **0.28** | **0.18** | **0.43** | **0.31** | **0.20** | **0.49** |
| North America | 17 | 0.3% | 1.11 | 0.69 | 1.78 | 1.27 | 0.79 | 2.04 |
| South America | 60 | 1.0% | 1.12 | 0.87 | 1.44 | 1.28 | 1.00 | 1.66 |

**Bold** denotes p<0.05

HR: Hazard ratio; 95%CI: 95% confidence interval

^1^Adjusted for age, sex, and time period

**Supplemental Table 6: Unadjusted and adjusted hazard ratios by Age-At-Migration, Sensitivity analysis with washout period**

|  |  |  | Unadjusted | |  | Adjusted^1^ | |  |
| --- | --- | --- | --- | --- | --- | --- | --- | --- |
|  | N | % | Hazard ratio | 95% CI |  | Hazard ratio | 95% CI |  |
| **Schizophrenia + schizoaffective** |  |  |  |  |  |  |  |  |
| Swedish born (reference) | 1,351 | 63.7% | 1 |  |  | 1 |  |  |
| Infancy | 49 | 2.3% | **1.56** | **1.17** | **2.09** | **1.57** | **1.18** | **2.10** |
| Early childhood | 89 | 4.3% | **2.15** | **1.73** | **2.67** | **2.15** | **1.73** | **2.66** |
| Middle childhood | 132 | 6.3% | **2.34** | **1.96** | **2.80** | **2.33** | **1.94** | **2.79** |
| Adolescence | 86 | 4.1% | **2.48** | **2.00** | **3.09** | **2.48** | **1.96** | **3.14** |
| Early adulthood | 36 | 1.7% | 1.46 | 1.04 | 2.04 | 1.81 | 0.89 | 3.71 |
| **Affective psychotic disorders** |  |  |  |  |  |  |  |  |
| Swedish born (reference) | 1,532 | 73.2% | 1 |  |  | 1 |  |  |
| Infancy | 58 | 2.8% | **1.69** | **1.30** | **2.21** | **1.71** | **1.31** | **2.24** |
| Early childhood | 58 | 2.8% | 1.27 | 0.97 | 1.66 | 1.29 | 0.99 | 1.68 |
| Middle childhood | 65 | 3.1% | 1.07 | 0.83 | 1.37 | 1.13 | 0.88 | 1.45 |
| Adolescence | 64 | 3.1% | **1.58** | **1.23** | **2.04** | **1.61** | **1.24** | **2.09** |
| Early adulthood | 57 | 2.7% | **1.42** | **1.08** | **1.86** | **1.47** | **0.74** | **2.90** |
|  |  |  |  |  |  |  |  |  |
| **Other non-affective psychotic disorders** |  |  |  |  |  |  |  |  |
| Swedish born (reference) | 2,899 | 66.6% | 1 |  |  | 1 |  |  |
| Infancy | 126 | 2.9% | **1.92** | **1.60** | **2.30** | **1.95** | **1.63** | **2.34** |
| Early childhood | 191 | 4.4% | **2.15** | **1.86** | **2.49** | **2.15** | **1.86** | **2.49** |
| Middle childhood | 213 | 4.8% | **1.75** | **1.52** | **2.02** | **1.82** | **1.58** | **2.10** |
| Adolescence | 162 | 3.7% | **2.08** | **1.78** | **2.44** | **1.93** | **1.63** | **2.29** |
| Early adulthood | 79 | 1.8% | 1.14 | 0.91 | 1.43 | 0.73 | 0.44 | 1.22 |
| **Bipolar disorder without psychosis** |  |  |  |  |  |  |  |  |
| Swedish born (reference) | 5,130 | 82.1% | 1 |  |  | 1 |  |  |
| Infancy | 141 | 2.3% | **1.19** | **1.00** | **1.40** | **1.20** | **1.01** | **1.42** |
| Early childhood | 83 | 1.3% | **0.53** | **0.42** | **0.66** | **0.54** | **0.43** | **0.67** |
| Middle childhood | 97 | 1.6% | **0.45** | **0.36** | **0.55** | **0.49** | **0.40** | **0.59** |
| Adolescence | 36 | 0.6% | **0.27** | **0.19** | **0.37** | **0.26** | **0.18** | **0.36** |
| Early adulthood | 37 | 0.6% | **0.33** | **0.24** | **0.46** | **0.28** | **0.14** | **0.56** |

**Bold** denotes p<0.05

HR: Hazard ratio; 95%CI: 95% confidence interval

^1^Adjusted for age, sex, and time period

**Supplemental Table 7: Test of proportional hazards assumption**

| Outcome | Exposure | Chi^2^ | df | p |
| --- | --- | --- | --- | --- |
| Schizophrenia + schizoaffective disorder | Migrant status | 11.49 | 10 | 0.32 |
|  | Region of origin | 21.08 | 18 | 0.28 |
|  | Age-at-migration | 16.99 | 14 | 0.26 |
| Affective psychotic disorders | Migrant status | 20.01 | 10 | 0.03 |
|  | Region of origin | 35.00 | 18 | 0.01 |
|  | Age-at-migration | 24.22 | 14 | 0.04 |
| Other non-affective psychotic disorders | Migrant status | 48.16 | 10 | <0.01 |
|  | Region of origin | 51.98 | 18 | <0.01 |
|  | Age-at-migration | 57.09 | 14 | <0.01 |
| Bipolar disorder without psychosis | Migrant status | 75.65 | 10 | <0.01 |
|  | Region of origin | 90.11 | 18 | <0.01 |
|  | Age-at-migration | 68.92 | 14 | <0.01 |

**Supplemental Figure 1: Scaled residual plots for migrant status in relation to “other non-affective psychotic disorders” and bipolar disorder without psychosis**

**C.**

**D.**

**A.**

**B.**

**Legend**: Figures A-D show log-log plots of Schoenfeld residuals for migrants and their children with respect to “other non-affective psychotic disorders” (**A, B**) and bipolar disorder without psychosis (**C, D**), respectively. Residuals are taken from full Cox proportional hazards models, fitted with age, sex, migrant status, and time period. Although global tests suggested violation of proportional hazards (see Supplemental Table 7), inspection of these plots suggested little departure from zero slopes for residuals (pink line) over time in respect to migrant status, indicating proportional hazards were not violated for this variable. Residual plots for region of origin and age-at-migration with respect to these two outcomes showed similar patterns (data available from authors).
